# Root Exudates Metabolic Profiling Suggests Distinct Defense Mechanisms Between Resistant and Susceptible Tobacco Cultivars Against Black Shank Disease

**DOI:** 10.3389/fpls.2020.559775

**Published:** 2020-09-10

**Authors:** Chengsheng Zhang, Chao Feng, Yanfen Zheng, Jing Wang, Fenglong Wang

**Affiliations:** ^1^ Marine Agriculture Research Center, Tobacco Research Institute of Chinese Academy of Agricultural Sciences, Qingdao, China; ^2^ Qingdao Special Crops Research Center of Chinese Academy of Agricultural Sciences, Qingdao, China; ^3^ Pest Integrated Management Key Laboratory of China Tobacco, Qingdao, China

**Keywords:** *Phytophthora nicotianae*, metabolic profiling, tobacco, *Nicotiana*, disease resistance, defense mechanisms

## Abstract

There is increasing evidence that root exudates play important roles in plant disease resistance. Black shank, caused by *Phytophthora nicotianae*, is a destructive soil-borne disease in tobacco (*Nicotiana tabacum* L.). The aim of the present study was to investigate the activity and composition of the root exudates from resistant and susceptible tobacco cultivars. The root exudates of the resistant cultivar Gexin 3 showed inhibitory activity against *P. nicotianae*, while the exudates of susceptible cultivar Xiaohuangjin 1025 stimulated the colony growth but had no effect on spore germination. Metabolic profiling using liquid chromatography/electrospray ionization-quadrupole-time-of-flight mass spectrometry depicted differing metabolic patterns of root exudates between Gexin 3 and Xiaohuangjin 1025. The activity and composition of root exudates was altered by *P. nicotianae* inoculation. Multivariate analysis showed that root exudates (including organic acids, alkaloids, fatty acids, and esters) were different between the two varieties. The defense substances in root exudates, such as tartaric acid, ferulic acid, and lauric acid, may represent a pre-infection prevention strategy for tobacco. Phenylpropanoids as well as inducers of salicylic acid, fatty acids, 6-hydroxyhexanoic acid, and hydrojasmonate may be involved in tobacco defense responses. Compared to the susceptible cultivar, the roots of the resistant cultivar exhibited high enzyme activities of phenylalanine ammonia-lyase, cinnamate-4-hydroxylase and 4-coumarate-CoA ligase, which may prompt the synthesis and secretion of phenylpropanoids. Our results indicated that the root exudates not only provide a pre-infection prevention strategy by exuding antimicrobial substances, but also increase tobacco disease resistance by eliciting plant defense responses. In addition, some defense compounds as well as compounds that play a role in inducing plant defense responses, showed potential for disease control application. This study provides an insight into possible disease resistance mechanisms of root exudates, and attempts the beneficial utilization of these secondary metabolites of plants.

## Introduction

*Phytophthora nicotianae* Breda de Haan (syn. *P. parasitica* Dastur) is a typical soil borne pathogen with great economic significance and academic research value (Panabières et al., 2016). It can infect 255 species of plants in 90 families, and cause serious disease in a variety of crops, such as tobacco (*Nicotiana* spp.), tomato (*Lycopersicum esculentum*), and citrus (*Citrus* spp.) (Cline et al., 2008). Because of long surviving resting structures (oospores and chlamydospores), coupled with hidden initial infection sites, it is very difficult to prevent. The use of host resistance has proved to be the most economical, effective, and environmentally-friendly strategy for the control of disease; knowledge about these mechanisms can benefit the development of approaches to disease control and prevention (Wang et al., 2018).

Plants have evolved complex defense systems during long-term interactions with pathogens (Dodds and Rathjen, 2010). Recently, there is increasing evidence that root exudates play important roles in plant resistance against biotic and abiotic stress (reviewed by Coninck et al., 2015; Tsunoda and van Dam, 2017; Canarini et al., 2019). However, the significance of root exudates associated with plant disease resistance has long been underestimated. Previous studies have indicated that root exudates of resistant and susceptible crop cultivars had different effects on pathogens (Whalley and Taylor, 1973; Stevenson et al., 1995). Root exudates of some resistant varieties had an inhibitory effect on pathogen growth, whereas those of some susceptible varieties exhibited a stimulatory effect (Wu et al., 2008; Schalchli et al., 2012), indicating a relationship between the activity of root exudates and plant disease resistance.

There are distinct differences between cultivars with respect to the effect of root exudate composition on pathogen activity. Therefore, deciphering the chemical profiles in exudates is important to understanding plant–pathogen interactions belowground. However, it is very difficult to collect and identify the components of plant root exudates because of their low content and complex composition, and their metabolism being affected by many factors. Thus, the lack of comprehensive knowledge on exudate chemistry represents a major bottleneck in the understanding of “plant–pathogen interactions” in the rhizosphere (Vives-Peris et al., 2019). Benefiting from the recent advances in metabolomics and multivariate analysis, liquid and gas chromatography platforms have successfully been used for profiling of root exudates (van Dam and Bouwmeester, 2016; Tsunoda and van Dam, 2017; Gfeller et al., 2018). In this study, tobacco-*P. nicotianae* interaction was selected as the host-pathogen research system. We systematically investigate the activity and composition of root exudates from a resistant and a susceptible cultivar. The associations between the presence of particular root exudates and disease resistance are analyzed. Moreover, some components with potential application in disease control are evaluated. The results provide an insight into the plant defense strategies mediated by root exudates.

## Material and Methods

### Materials

The highly susceptible tobacco cultivar “Xiaohuangjin 1025” (S) and the highly resistant cultivar “Gexin 3” (R) were obtained from the National Medium-term Genebank of Tobacco Germplasm Resource of China. *P. nicotianae* race 0 strain JM01 with high pathogenicity was obtained from the Integrated Pest Management Key Laboratory of China Tobacco, Qingdao, China. All reagents used in this study were purchased from Sigma Chemical Co. (St. Louis, MO, USA).

### Preparation of Swimming Spores of *P. nicotianae*

The zoospores of *P. nicotianae* were prepared according to the method described by Zhang et al. (2017). Briefly, *P. nicotianae* were cultured for 21 days on oatmeal agar (OA) in a Petri dish. Then, 0.1% KNO_3_ solution was added (10 ml per dish), followed by culturing at 26°C for 72 h, and immediately chilled to 4°C for 0.5 h. Spore suspension was carefully drawn, adjusted to 10^6^ colony-forming units (cfu) ml^-1^, and stored at -4°C.

*P. nicotianae* for field inoculation were prepared by culturing on a medium prepared from millet. The millet seed was boiled until half of the seed coat ruptured. The seed was then filtered through gauze, placed in a conical flask, and sterilized for 20 min at 121°C. After cooling, a cake of *P. nicotianae* was transferred to the millet medium, cultured at 26°C for 15 d, and used within 24 h.

### Identification of Disease-Resistant Tobacco Varieties

The tobacco black shank resistance of the S and R cultivars was evaluated with disease nursery artificial induction in the field and the greenhouse. Tobacco plants (R and S) with five true leaves cultured in sterilized potting soil (10 cm × 10 cm) were used for the greenhouse experiment. Each plant was irrigated with 10 ml of *P. nicotianae* spore suspensions (10^6^ cfu ml^-1^). After incubating at 28°C for 7 days (relative humidity 70%, light intensity 300 μmol m^−2^ s^−1^, 12 h light/12 h dark), the disease severity was recorded, and the disease index was calculated based on the method described by Zhang et al. (2017). In the control treatment, plants were irrigated with 10 ml of distilled water. Each treatment repeated three times, and every replicate had 15 seedlings for each cultivar.

Field experiments were conducted in 2017 in a disease nursery of tobacco black shank located at Jimo Tobacco Resources and Environmental Field Experiment Station of the Chinese Academy of Agricultural Sciences (36°27′N, 120°35′E). Tobacco seedlings were planted in early-June, and inoculated with millet containing *P. nicotianae* in early-July. The disease severity and the disease index were assessed in early-August using the method described by Han et al. (2016), when the tobacco plants had reached a mature stage. Each treatment was repeated 3 times with each replicate containing about 45 tobacco plants (three rows, 15 plants per row).

### Root Exudate Collection

Tobacco plants were treated according to the greenhouse experiment method described above, and root exudates were collected when the plants had eight true leaves. The treatments included: resistant variety (R), susceptible variety (S), resistant variety inoculated with *P. nicotianae* (Ri), and susceptible variety inoculated with *P. nicotianae* (Si). Inoculated plants were sampled at the third day after inoculation. The seedlings were carefully dug up, the soil attached to the root surface was washed away with tap water, and the roots were washed three times with distilled water. Three seedlings were put in a conical flask (500 ml), and the roots immersed in 300 ml of distilled water. After incubating for 6 h under aeration conditions, the culture solution was freeze-dried, weighed, and transferred to a test tube with 10 ml of precooled methanol, and stored at -80°C. The yield of root exudates was defined as the ratio of root exudates to root dry weight. Each treatment was repeated six times.

### Effect of Root Exudates on Mycelium Growth and Zoospore Germination of *P. nicotianae*

The mycelial growth and zoospore germination assays of root exudates obtained from R, S, Ri, and Si were performed according to the method described by Zhang et al. (2017). Root exudates were dissolved in dimethyl sulfoxide (DMSO), filtered with microporous membrane (0.22 μm), and added to OA plates (Φ=9 cm) at a final concentration of 0.05 g ml^-1^. Then, each dish was inoculated with a *P. nicotianae* mycelium cake (with diameter of 5 cm), and incubated at 26°C in the dark. The diameter of mycelium was determined on the third day. An equal volume of sterilized distilled water served as a control. Each treatment had five replicates.

Then, 100 μl of spore suspension (diluted to 10^3^ cfu ml^-1^) was placed on a concave slide, blended with an equal volume of root exudate with a final concentration of 0.05 g ml^-1^, and incubated for 10 h at 26°C in the dark with relative humidity of 80%. Spore germination was recorded as a percentage of the total zoospores by using a light microscope and a hemocytometer. An equal volume of sterilized distilled water served as a control. The relative inhibitory rate (RIR) was calculated according to the following equation.

$$\text{RIR}=\frac{\left(\text{Germination}\;\text{rate}\;\text{of}\;\text{control}-\text{Germination}\;\text{rate}\;\text{of}\;\text{treatment}\right)}{\text{Germination}\;\text{rate}\;\text{of}\;\text{control}}\times 100$$

### Liquid Chromatography-Mass Spectrometry Analysis of Root Exudates

Freeze-dried root exudates (200 mg) were suspended in 5 ml of 0.1% HCOOH in 80% methanol, filtered through a 0.22-μm cellulose membrane and transferred to an amber vial for analysis. Samples (4 μl) were analyzed using ultra-high-performance liquid chromatography coupled with electrospray ionization quadrupole-time-of-flight mass spectrometry (UHPLC/ESI-Q-TOF-MS) (Ultimate 3000 LC, Orbitrap Elite, Thermo Fisher, America). The chromatographic separation was performed on a Hypergod C_18_ (length × internal diameter × Silica gel: 100 mm × 4.6 mm × 3 μm) column. The mobile phase was 0.1% formic acid-water (A) and acetonitrile with 0.1% formic acid (B). The gradient conditions of the mobile phase were as follows: 0–1 min, 95% A; 1–6 min, 95–80% A; 6–9 min, 80–50% A; 9–13 min, 50–5% A; 13–15 min, 5% A.

Each sample (4 µl) was injected in random order at 4°C, and the analysis was performed in both positive and negative ion modes. In positive ion mode, the dissolution gas temperature was 350°C and the flow rates of sheath gas, aux gas, and sweep gas were 45, 15, and 1 arb, respectively. The mass spectra were acquired using electrospray ionization (ESI) in negative and positive ionization modes. The mass scanning range was 50–1,000 m/z. The scan time of 0.03 s was used throughout the experiment, with interval scan time of 0.02 s. The spray voltage was 3.0 kV and the S-Lens RF Level was 30% in positive ion mode. In negative ion mode, the spray voltage was 3.2 kV and the S-Lens RF Level was 60%. To ensure accuracy and reproducibility, leucine–enkephalin was used as the lock mass for positive [M+H]^+^ (m/z 556.2771) and negative [M-H]^−^ (m/z 554.2615) ion modes.

To assess the reproducibility and reliability of the LC/ESI-Q-TOF-MS system, quality control (QC) samples were also prepared by mixing equal volumes (10 μl from each sample of root exudate. The pooled QC sample was injected six times at the beginning of the run to ensure system equilibrium and then after every 6 samples to further monitor the stability of the analysis (Luan et al., 2014; Zhou et al., 2016).

### UHPLC/ESI-Q-TOF-MS Data Processing and Analysis

The original data collected by mass spectrometry were processed using sievev2.1 software (Thermo Fisher, MA, USA), including noise filtering, overlapping peak analysis, peak alignment, peak matching, standardization, and normalization. The peak table output from positive and negative ion were saved as Excel tables. Thereafter, features consistently detectable in at least 75% of the replicate exudate samples were screened. Post-acquisition processing included compound filtering by abundance (area > 5,000 counts), normalization at the 75th percentile, and baselining to the median of the control. Unsupervised hierarchical cluster analysis was then carried out on the basis of fold-change-based heatmaps, setting the similarity measure as “Euclidean” and using “Wards” as the linkage rule.

Then, the resulting three-dimensional data matrix containing m/z-retention time pairs, sample names, and their normalized chromatographic peak areas (variables) were exported into SIMCA-P 11.0 (Umetrics, Sweden) for supervised orthogonal partial least squares discriminant analysis (OPLS-DA). OPLS-DA score plots were used to evaluate the quality of the model by the relevant parameters R^2^ and Q^2^. The differential metabolites were selected when the statistically significant threshold of variable importance in projection (VIP) values obtained from the OPLS-DA model was > 1.0. Furthermore, the *p* values from a two-tailed Student’s t-test on the normalized peak areas were < 0.05. Log_2_-fold-change was used to show how these selected differential metabolites varied between groups.

The screened discriminant compounds were tentatively annotated by comparing the measured relative molecular mas with the theoretical value (http://metlin.scripps.edu/). For better visualization of the metabolic signature among the four groups, the top 22 representative candidates (VIP > 1.5) were screened according to ANOVA for clearer thermographic visualization. The heatmap was generated using TB Tools software (Chen et al., 2020).

### Key Enzyme Activities in Phenylpropanoid Metabolism

On days 0, 1, 2, 3, 4, and 5 after inoculation, fresh roots were sampled and used to determine activity of three enzymes involved in phenylpropanoid metabolism according to Fan et al. (2012): phenylalanine ammonia-lyase (PAL), cinnamate-4-hydroxylase (C4H), and 4-coumarate-CoA ligase (4CL).

A sample of 3 g of roots was ground with 3 ml of 0.1 mol l^-1^ boracic acid buffer [pH 8.8, containing 10% (w/v) PVPP, 1 mmol l^-1^ EDTA, and 50 mmol l^-1^ β-mercaptoethanol], followed by centrifugation for 30 min (15,000 g) at 4°C. The supernatant was collected as crude enzyme and used to determine PAL activity. The reaction system included 2 ml of 0.02 mmol l^-1^ L-phenylalanine (prepared with boracic acid buffer) and 200 μl of crude enzyme of PAL. The mixture was incubated for 30 min at 30°C, and 200 μl of 6 mol l^-1^ HCL was added to terminate the reaction. Optical density (OD) values were measured at a wavelength of 290 nm.

Crude C4H was extracted by grinding 3 g of tobacco root with 5 ml of extraction solution containing 50 mmol l^-1^ Tris-HCl, 15 mmol l^-1^ (pH 8.9), β-mercaptoethanol, 4 mmol l^-1^ MgCl_2_, 5 mmol l^-1^ Vc, 10 μmol l^-1^ leupeptin, 1 mmol l^-1^ PMSF, 0.15% (w/v), and 10% glycerol, and centrifuged for 20 min (12,000 g). The supernatant was collected as crude C4H. The enzyme activity was assayed with the following steps: 0.8 ml of crude enzyme mixed with 2.2 ml of buffer (2 μmol l^-1^ trans-cinnamic acid, 50 mmol l^-1^ Tris-HCl (pH 8.9), 2 μmol l^-1^ NADPNa_2_, 5 μmol l^-1^ G-6-pNa). OD values were measured at a wavelength of 620 nm.

For the crude 4CL extraction, 3 g of tobacco root were ground with 3 ml of 0.2 mol l^-1^ Tris-HCl (containing 25% glycerol and 0.1 mol l^-1^ DTT, pH 8.0) and a small amount of quartz sand and centrifuged for 20 min (15,000 g). The supernatant was collected. The reaction system for 4CL activity determination included: 0.45 ml of 15 μmol l^-1^ Mg ^2+^, 0.15 ml of 5 μmol l^-1^ p-coumaric acid, 0.15 ml of 5 μmol l^-1^ ATP, 0.15 ml of 1 μmol l^-1^ CoA, and 0.5 ml of crude enzyme. After 10 min of reaction at 40°C, the OD values were measured at a wavelength of 330 nm.

One enzyme activity unit (U) of PAL was defined as 0.01 of OD_290_ value change per hour, which was indicated as 1 U·h^-1^ g^-1^·FW. One U of C4H and 4CL was defined as 0.01 of OD_340_ and OD_333_ per minute, respectively, and was indicated as 1 U·min^-1^ g^-1^·FW.

The whole process of crude enzyme extraction was carried out at 4°C. Distilled water (equal volumes to the enzyme mixture) served as a control. Each treatment was repeated three times.

### Effects of Compounds on Mycelium Growth of *P. nicotianae*

Twelve compounds were selected from the representative candidates for further study on *P. nicotianae* mycelial growth, including three organic acids (SA, tartaric acid, and ferulic acid), two esters (glycerol tripropanoate, isoamyl cinnamate), three fatty acids (linolenic acid, oleic acid, and lauric acid), and one alcohol (abietinol), ketone (prohydrojasmon), and terpenoid (casbene). These compounds are known as potential allelochemicals. The compounds tested were dissolved by DMSO, diluted to the required concentration as follows with ultrapure water, and filtered with 0.22 µm millipore filtration membrane. The prepared compound solutions were added to OA medium plate to ensure the final concentration as 5, 50, and 500 µg ml^-1^, respectively. Then, a cake of *P. nicotianae* (0.6 cm diameter) was placed in the center of the plate, and incubated in dark at 26°C for 5 days. The colony diameter was measured by the cross method, and the inhibition rate of mycelium growth were calculated (Zhang et al., 2017). The DMSO served as the control. Each treatment had three replicates, and each replicate had three plates.

### Pot Experiment of Tobacco Black Shank Control

To evaluate the potential of compounds for control of *P. nicotianae*, a pot experiment was carried out according to the method described in section 2.3. Seedlings of Xiaohuangjin 1025 (with five true leaves) were transplanted to pots (one plant per pot), and the seedlings were irrigated with 10 ml of ferulic acid (500 µg ml^-1^), 10 ml of lauric acid (500 µg ml^-1^), and 10 ml of salicylic acid (SA, 100 µg ml^-1^). Our preliminary experimental results showed that a high concentration of SA (> 150 μg ml^-1^) is harmful to tobacco seedlings. Therefore, the test concentration of SA was set at 100 µg ml^-1^. After treatment for 24 h, plants were inoculated with *P. nicotianae* by irrigating each plant with 10 ml of zoospores (10^6^ cfu ml^-1^). Thereafter, the seedlings were incubated at 28°C and 70% relative humidity. The disease severity was assessed on the fifth day and tenth day after inoculation using the method described by Han et al. (2016). Each compound was diluted with 2% DMSO. For the control, each compound was diluted with the same amount of distilled water. Each treatment consisted of 15 seedlings with three replicates.

### Data Statistics

Excel 2013 and DPS 7.05 software were used for data analysis. Differences between groups were tested using one-way ANOVA, followed by Tukey’s multiple comparison test. *P* < 0.05 indicates that the differences are statistically significant.

## Results

### Disease Resistance of Two Tobacco Varieties

**Figure 1:**
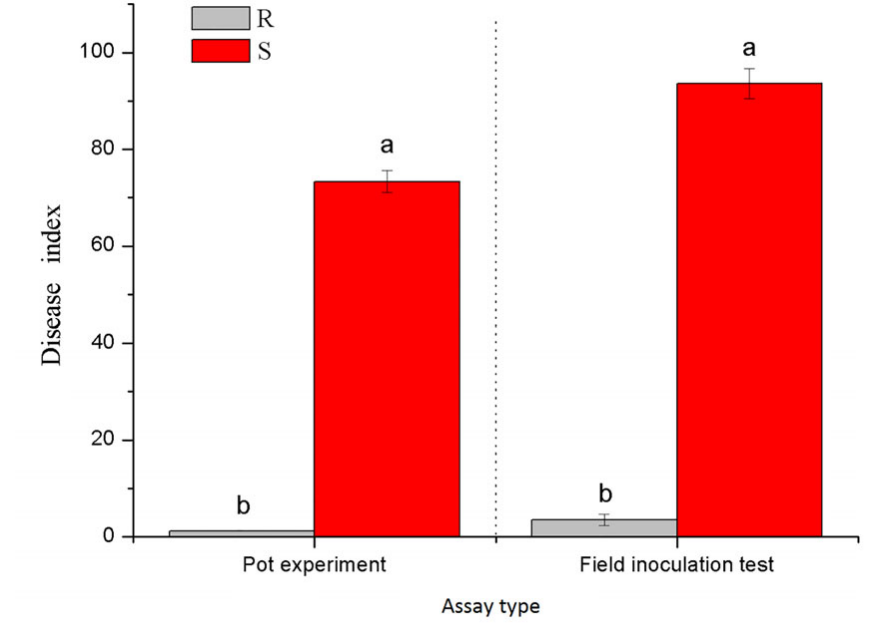
Resistance identification of tobacco cultivars against black shank under pot (a) and field (b) conditions. R and S represent resistant and susceptible cultivars, respectively; different lowercase letters indicate statistical differences between groups at *P* < 0.05; the error bars represent standard error of the mean for n = 3.

Xiaohuangjin 1025 (S) had a disease index of 73.33 in the pot experiment (**Figure S1A**) and 93.58 in the field experiment; Gexin 3 (R) showed a slight occurrence of black shank with a disease index of 1.25 in the pot experiment (**Figure S1B**) and 2.39 in the field experiment (**Figure 1**). These results verified that Gexin 3 is highly resistant to tobacco black shank both at the seedling stage and at the adult stage, while Xiaohuangjin 1025 is highly susceptible.

### Root Dry Weight and Exudate Yield of Different Varieties

**Figure 2:**
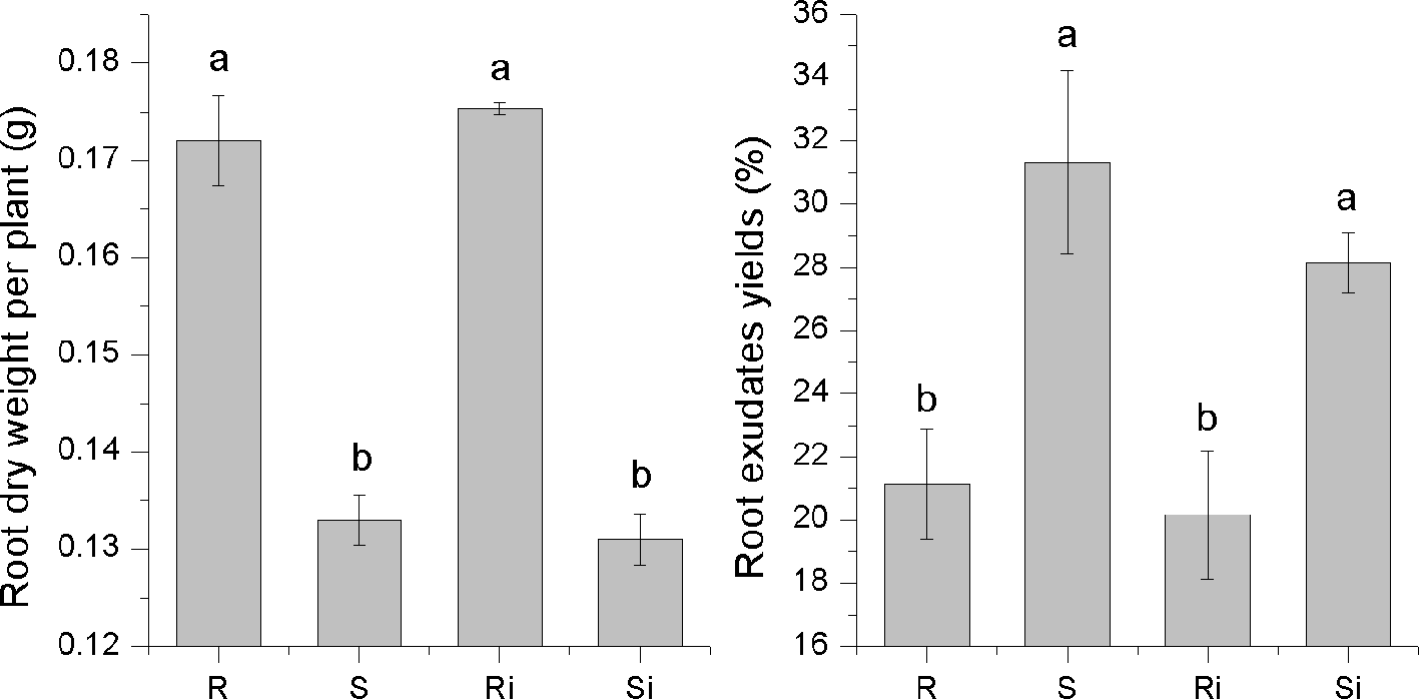
The dry root weight and exudate yields of tobacco cultivars. Ri and R represent inoculated and non-inoculated plants of the resistant cultivar, respectively; Si and S represent inoculated and non-inoculated plants of the susceptible cultivar, respectively; different lowercase letters indicate statistical differences between groups at *P* < 0.05; the error bars represent standard error of the mean for n = 6.

A significant difference was observed in the secretion of root exudates between the two varieties (**Figure 2**). The root exudate yield of cultivar S (31.32%) was significantly higher than that of cultivar R (21.12%) (*P* < 0.05). However, the root dry weight of R (0.17 g per plant) was significantly higher than that of S (0.13 g per plant) (*P* < 0.05), which led to no significant difference in the amount of root exudate per plant between the two varieties. The inoculation of *P. nicotianae* caused a decrease in root exudate yield of both S and R, but the difference was not significant.

### Effects of Root Exudates on *P. nicotianae*

**Figure 3:**
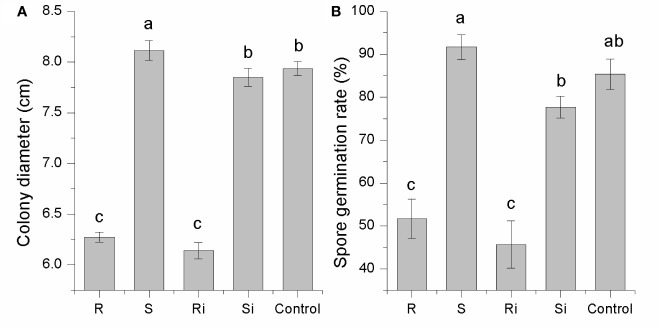
Inhibitory effects of root exudates on mycelial growth **(A)** and spore germination **(B)** of *P. nicotianae*. Ri and R represent inoculated and non-inoculated plants of the resistant cultivar, respectively; Si and S represent inoculated and non-inoculated plants of the susceptible cultivar, respectively; different lowercase letters indicate statistical differences between groups at *P* < 0.05; the error bars represent standard error of the mean for n = 3.

As shown in **Figure 3**, the effects of root exudates on mycelial growth and spore germination were distinct between cultivar R and cultivar S. Root exudates from healthy or infected plants of R displayed a strong inhibitory effect on both mycelial growth (30–46%) and spore germination (25–40%). The effect of root exudates from S differed significantly between healthy and infected plants, i.e., there was a significant stimulatory effect (7–14%) from exudates of healthy plants (*P* < 0.05), while the exudates of infected plants showed a slight inhibitory effect (< 5%). These results indicated that *P. nicotianae* infection led to changes in the root exudates of S plants, thus producing different effects on *P. nicotianae*.

### Profiling of Root Exudates With UHPLC/ESI-Q-TOF-MS

To evaluate the repeatability of the metabolite extractions and detections, namely their technical repeatability, an overlay analysis was performed to the TIC plots of different QC samples. **Figure S2A** shows an overlay of the TIC plots between QC samples (six samples at the beginning, one QC after the first six samples, and one QC after the last six samples) under ESI positive mode, while **Figure S2B** exhibits that between QC samples (the first and last QC samples) under ESI negative mode. The retention time and peak area of all QC samples had a high degree of overlap, indicating good QC-sample repeatability and instrumental stability. Therefore, the data recorded in this study showed good repeatability and reliability.

**Figure 4:**
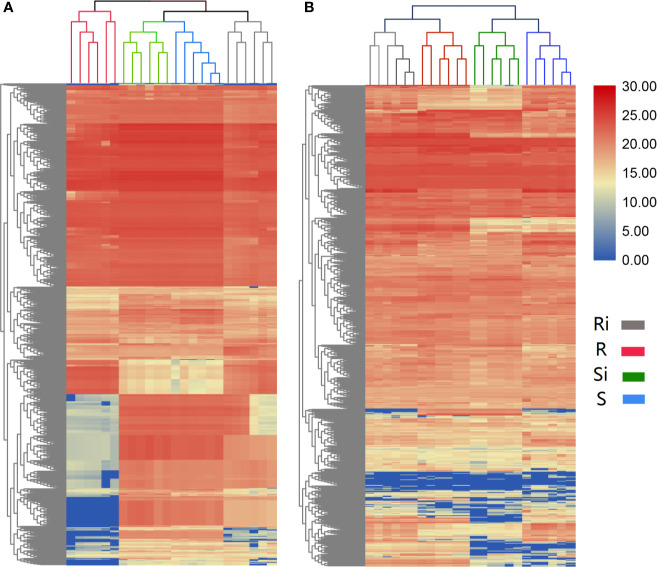
Unsupervised hierarchical cluster analysis (with Euclidean distance and Ward’s linkage rule) performed from root exudate chemical profile under positive **(A)** and negative **(B)** ion mode. R, non-inoculated Gexin 3; S, non-inoculated Xiaohuangjin 1025; Ri, inoculated Gexin 3; Si, inoculated Xiaohuangjin 1025. The numerical values for the blue-to-red gradient bar represent log_2_ peak area.

The results of UHPLC/ESI-Q-TOF-MS analysis showed that the chemical diversity of the root exudates was wide. In total, 685 peaks of positive ions and 873 peaks of negative ions were observed in UHPLC, and were used for further statistical analysis. The hierarchical clustering showed different clustering results between the two ESI ion modes. In both positive (**Figure 4A**) and negative (**Figure 4B**) ion mode, 24 samples from different treatments were clustered into 4 different groups, and each cluster contained all repeats within a treatment. In the positive ion mode of ESI, the clustering relationship of Xiaohuangjin 1025 inoculated (Si) and non-inoculated (S) with *P. nicotianae* was closer, while that of the non-inoculated treatment of Gexin 3 (Ri) was relatively independent of the other three treatments. In ESI negative ion mode, samples from the same cultivar (including inoculated and non-inoculated plants) showed higher similarity, indicating that root exudate metabolites were more affected by plant genotypes than pathogen infection. Hierarchical cluster analysis (HCA) revealed significant differences in root exudate patterns between R and S.

**Figure 5:**
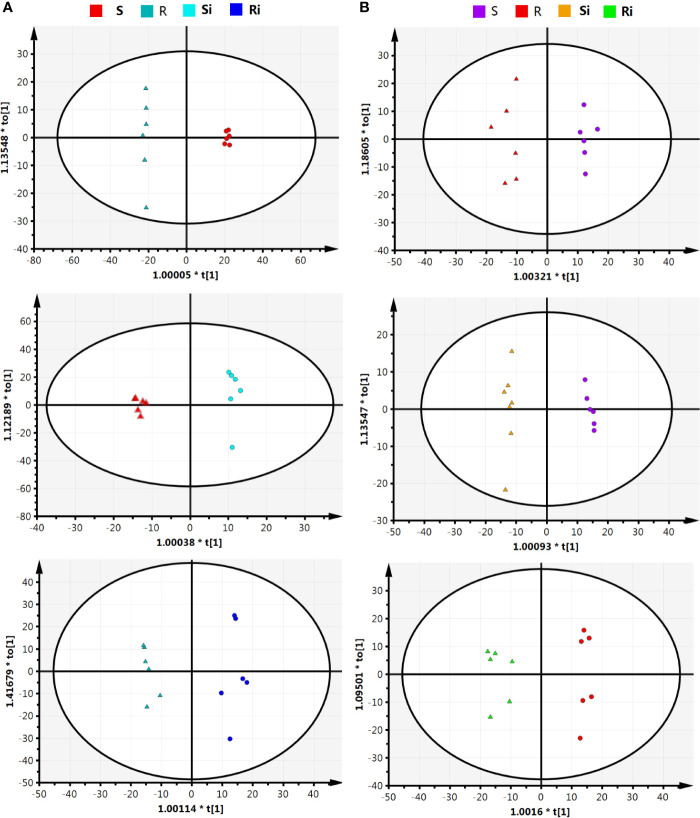
Orthogonal partial least squares discriminant analysis (OPLS-DA) score plot on root exudate profiles gained through UHPLC-ESI/QTOF-MS analysis under condition of positive **(A)** and negative **(B)** ion mode. R, non-inoculated Gexin 3; S, non-inoculated Xiaohuangjin 1025; Ri, inoculated Gexin 3; Si, inoculated Xiaohuangjin 1025.

The parameters of OPLS-DA models based on UHPLC/ESI-Q-TOF-MS are listed in **Table 1**. The cumulative (cum) values of R^2^Y and Q^2^ indicate the fitness and the prediction ability of the models, respectively. The closer the values are to 1, the more stable and reliable the model. The ranges of R^2^X (cum) and R^2^Y (cum) were 0.41–0.87 and 0.972–0.999, respectively, indicating that the variable differences between the two groups could be reflected by more than 41% of UHPLC/ESI-Q-TOF-MS data. Q^2^ (cum) was higher than 0.8, indicating that the model had better prediction. **Figure 5** shows that the OPLS model could clearly distinguish the samples in different groups, indicating that the root exudate patterns varied between the susceptible and resistant cultivars as well as between inoculated and non-inoculated plants.

**Table 1:** Parameters of OPLS-DA models based on UHPLC/ESI-Q-TOF-MS.

Sample comparison group	Ion mode	Number of principal components (principal + orthogonal)	R^2^X (cum *)	R^2^Y (cum)	Q^2^ (cum)	VIP value > 1 variables no.
R vs. S	+	2 + 1	0.752	0.999	0.982	46
R vs. S	–	1 + 1	0.473	0.965	0.847	30
S vs. Si	+	4 + 1	0.869	0.994	0.917	29
S vs. Si	–	1 + 1	0.411	0.994	0.882	45
R vs. Ri	+	3 + 1	0.819	0.974	0.824	10
R vs. Ri	–	1 + 1	0.585	0.972	0.904	43

*cum, cumulative.

### Screening of Potential Biomarkers in Exudates

**Table 1** shows the potential biomarkers in positive and negative ion revealed by variable importance in projection (VIP) analysis. Seventy-five, 52, and 72 discriminant compounds were screened in R vs S, R vs Ri and S vs Si, respectively, of which 45, 9, and 29 were found in ESI positive ion mode and 30, 43, and 43 in ESI negative ion mode. The 22 compounds with the highest discrimination potential (with a VIP score > 1.5 in at least one model) are listed in **Table 2**; the list includes four organic acids, three esters, seven alkaloids, four fatty acids, one alcohol, one ketone, one alkene, and one phenol. The negative ion mode revealed 15 compounds (mainly organic acids, alkaloids, and fatty acids), while the positive ion mode revealed seven compounds (mainly alkaloids, esters, and alcohols). Fold-change analysis was used to evaluate the regulation of these compounds, where log-fold changes > 1 and ≤ 1 were conceived as upregulation and downregulation, respectively. Log-fold changes of between -1 and 1 were not considered to represent changes in regulation. There are 12 compounds listed in **Table 3** with an R/S > 1; 7 compounds with an R/S ≤ -1; and 3 compounds with an R/S of between -1 and 1. The accumulation of SA, esculetin, oleic acid, isoamyl cinnamate, casbene, and 6-gingerol was enhanced by *P. nicotianae* inoculation in both the resistant cultivar (Ri) and the susceptible cultivar (Si), while the accumulation of methyl 5-hydroxyferulate was suppressed, indicating that these compounds may be related to the response of tobacco to *P. nicotianae* infection. Protoanemonin and linolenic acid exhibited opposite regulation between Si and Ri. These compounds with distinct regulation as well as level of change may be associated with differences in disease resistance between Gexin 3 and Xiaohuangjin 1025.

**Table 2:** Annotation of discriminant metabolites in tobacco exudates between resistant (R) and susceptible (S) cultivars through UHPLC/ESI-Q-TOF-MS analysis, including inoculation with *P. nicotianae* (Ri and Si).

Compound	Ion mode	Retention time	Measured mass	Calculated mass	Formula	Mass error (ppm)
Tartaric acid	ESI-	12.0130	150.0171	150.0164	C_4_H_6_O_6_	4.67
Salicylic acid	ESI-	7.2374	138.0322	138.0317	C_7_H_6_O_3_	3.62
Ferulic acid	ESI-	6.5892	194.0584	194.0579	C_10_H_10_O_4_	2.58
Dehydrochorismic acid	ESI-	9.3485	224.0331	224.0321	C_10_H_8_O_6_	4.46
Glycerol tripropanoate	ESI+	11.1400	260.1259	260.126	C_12_H_20_O_6_	-0.38
Isoamyl cinnamate	ESI+	8.1833	218.1306	218.1307	C_14_H_18_O_2_	-0.46
Methyl 5-hydroxyferulate	ESI-	6.2199	210.0533	210.0528	C_10_H_10_O_5_	2.38
Rishitin	ESI-	9.7571	222.1624	222.162	C_14_H_22_O_2_	1.80
Protoanemonin	ESI+	11.5827	96.0207	96.0211	C_5_H_4_O_2_	-4.17
Horhammericine	ESI+	11.1696	368.1729	368.1736	C_21_H_24_N_2_O_4_	-1.90
Esculetin	ESI-	5.5484	178.0272	178.0266	C_9_H_6_O_4_	3.37
Lycaconitine	ESI-	10.5605	668.3271	668.3309	C_36_H_48_N_2_O_10_	-5.69
Olitorin	ESI-	10.0616	696.3339	696.3357	C_35_H_52_O_14_	-2.58
Nudicauline	ESI-	10.8287	710.3388	710.3415	C_38_H_50_N_2_O_11_	-3.80
Linolenic acid	ESI-	11.3836	278.2251	278.2246	C_18_H_30_O_2_	1.80
Lauric acid	ESI-	5.6513	200.1766	200.1776	C_12_H_24_O_2_	-5.00
6-Hydroxyhexanoic acid	ESI-	5.0280	132.0791	132.0786	C_6_H_12_O_3_	3.79
Oleic acid	ESI-	14.0680	282.2565	282.2559	C_18_H_34_O_2_	2.13
Abietinol	ESI+	12.8227	308.2709	308.2715	C_20_H_36_O_2_	-1.95
Prohydrojasmon	ESI-	9.9044	254.1887	254.1882	C_15_H_26_O_3_	1.97
Casbene	ESI+	13.5474	272.2499	272.2504	C_20_H_32_	-1.84
6-Gingerol	ESI+	10.6734	294.1829	294.1831	C_17_H_26_O_4_	-0.68

The annotation of metabolites was based on comparison of accurate mass to the Scripps database (with 5 PPM of tolerance). Mass error = (measured mass – calculated mass)/calculated mass × 1,000,000.

**Table 3:** Discriminant metabolites in tobacco root exudates were selected through OPLS-DA supervised multivariate statistics and thereafter subjected to fold-change analysis.

Compound	Non-inoculated plants			Inoculated with *P. nicotianae*					
	Log_2_ R/S	Change	*P* value	Log_2_ R/Ri	Change	*P* value	Log_2_ S/Si	Change	*P* value
Tartaric acid	-1.55	down	0.0007	-0.88	–	0.0359	0.21	down	0.4129
Salicylic acid	1.88	up	0.0003	-1.05	down	0.0001	-1.52	down	0.0023
Ferulic acid	1.65	up	0.0024	-0.64	–	0.0766	-2.37	down	<0.0001
Dehydrochorismic acid	0.44	–	0.0368	-0.76	–	0.0005	-1.14	down	0.0008
Glycerol tripropanoate	9.76	up	0.0001	-0.01	–	0.9701	-2.78	–	0.1148
Isoamyl cinnamate	-6.27	down	0.0007	-4.92	down	0.0007	-5.08	down	0.0073
Methyl 5-hydroxyferulate	-0.18	–	0.0404	2.45	up	<0.0001	1.86	up	<0.0001
Rishitin	3.29	up	0.0055	-0.76	–	0.0199	-3.69	down	<0.0001
Protoanemonin	-3.37	down	0.0003	-3.09	down	0.0002	1.78	up	0.0001
Horhammericine	-1.32	down	0.3167	-0.09	–	0.1542	1.81	up	0.0025
Esculetin	1.41	up	0.0057	-2.09	down	0.0004	-4.17	down	0.0026
Lycaconitine	6.85	up	<0.0001	0.49	–	0.0279	0.73	–	0.0844
Olitorin	5.29	up	<0.0001	0.28	–	0.0796	0.61	–	0.2540
Nudicauline	4.28	up	0.0002	0.50	–	0.0092	-1.13	–	0.0178
Linolenic acid	3.35	up	0.0002	2.12	up	<0.0001	-1.76	down	0.0002
Lauric acid	2.53	up	0.0172	-0.80	–	0.0012	-0.91	–	0.0193
6-Hydroxyhexanoic acid	2.08	up	0.0005	-0.62	–	0.0207	-1.14	down	0.0291
Oleic acid	0.45	–	0.0875	-2.03	down	<0.0001	-1.99	down	<0.0001
Abietinol	-5.69	down	0.0005	-0.47	–	0.3150	3.51	up	<0.0001
Prohydrojasmon	8.11	up	0.0002	0.73	–	0.0004	1.75	up	0.0497
Casbene	-1.33	down	0.0058	-0.09	down	0.7916	1.81	down	0.0025
6-Gingerol	-5.42	down	0.0049	-6.14	down	<0.0001	-4.66	down	0.0404

The annotation of metabolites was based on comparison of accurate mass to the Scripps database (with 5 PPM of tolerance). The significance of differences between two groups compared was determined with the Student’s t-test (n = 6). Results were considered significant at a 2-tailed P value of 0.05.

Analysis combined with hierarchical Pearson clustering was used to visualize the differences of metabolites in the four treatments. The top 22 representative candidates were screened according to ANOVA for clearer thermographic visualization. As shown in **Figure S3**, samples from the same treatment clustered together indicating that the differentially regulated compounds obtained have similar characteristics. This also illustrated that the metabolites of pathogen infected of plants (Ri and Si) varied from those of healthy plants (R and S). In particular, Si was separated from the other three groups. This may be related to the higher disease severity of Si compared to non-inoculated groups (R and S) and Ri (no obvious disease symptoms). R was richer in SA, ferulic acid, esculetin, prohydrojasmon, glycerol tripropanoate, lycaconitine, olitorin, nudicauline, linolenic acid, rishitin, and lauric acid compared to S, while S had higher contents of tartaric acid, protoanemonin, isomyl cinnamate, abietinol, and 6-gingerol. Isoamyl cinnamate, oleic acid, 6-gingerol, SA and esculetin were upregulated both in Ri and Si.

### Key Enzyme Activity in Phenylpropanoid Metabolism

**Figure 6:**
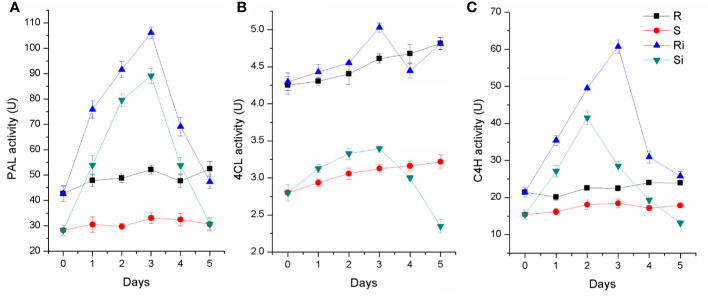
The activities of PAL **(A)**, 4CL **(B)** and C4H **(C)** in tobacco root of Gexin 3 and Xiaohuangjin 1025 inoculated with *P. nicotianae*. R, non-inoculated Gexin 3; S, non-inoculated Xiaohuangjin 1025; Ri, inoculated Gexin 3; Si, inoculated Xiaohuangjin 1025. The values are mean ± SE of three replicates for each treatment. Bars indicate SEs.

As shown in **Figure 6**, activities of PAL, C4H, and 4CL were higher in the roots of Gexin 3 than in those of Xiaohuangjin 1025. The enzyme activities were significantly affected by *P. nicotianae* inoculation (*P* < 0.05). The activities of PAL, C4H and 4CL increased significantly after inoculation, and peaked at the second or third day, followed by a quick descent. All three enzymes of Gexin 3 returned to a level close to the non-inoculated plants by the fifth day, and PAL activity of Xiaohuangjin 1025 also showed a similar trend. However, C4H and 4CL activities of Xiaohuangjin 1025 finally dropped to a level significantly lower than that of the non-inoculated plants (*P* < 0.05).

### Effect of Representative Compounds on *P. nicotianae* Mycelial Growth

As shown in **Table 4**, most of the compounds did not inhibit the growth of mycelium at a concentration of 5 µg ml^-1^. Three concentrations of glycerol tripropanoate, isoamyl cinnamate, linolenic acid, and oleic acid had no inhibitory effect, and there was even a stimulatory effect on the growth of *P. nicotianae*. Octadecanoic acid, abietinol, prohydrojasmon, and casbene showed low inhibitory activity with <50% inhibition rate after treatment with 500 µg ml^-1^. Tartaric acid, ferulic acid, and lauric acid showed strong inhibitory activity with inhibition rates of 87, 87, and 85% at 500 µg ml^-1^, respectively. SA also showed good inhibitory activity at 500 µg ml^-1^ (50%). Overall, organic acids and fatty acids showed a relatively higher inhibitory effect on *P. nicotianae* mycelial growth compared to esters, suggesting that these compounds are associated with the allelopathy of root exudates.

**Table 4:** Effect of tested compounds on mycelial growth of *P. nicotianae*.

Compound	Mycelial growth inhibition (%)		
	Treatment with 5 µg ml^-1^	Treatment with 50 µg ml^-1^	Treatment with 500 µg ml^-1^
Salicylic acid	0.16 ± 0.49 c	5.85 ± 0.76 d	50.36 ± 0.63 b
Tartaric acid	5.25 ± 0.72 a	22.12 ± 0.60 a	86.97 ± 0.91 a
Ferulic acid	5.73 ± 0.72 a	22.75 ± 0.47 a	87.05 ± 1.10 a
Glycerol tripropanoate	0.55 ± 0.72 c	1.26 ± 0.99 g	-1.12 ± 0.84 g
Isoamyl cinnamate	-0.16 ± 0.83 c	-0.95 ± 1.09g	-3.04 ± 0.73 g
Linolenic acid	-0.71 ± 0.47 c	-0.55 ± 0.49 g	3.04 ± 0.84 h
Oleic acid	0.31 ± 0.76 c	0.71 ± 0.85 g	0.08 ± 0.50 g
Lauric acid	0.24 ± 1.25 c	15.44 ± 1.31 b	85.37 ± 0.63 a
Octadecanoic acid	-0.95 ± 1.08 c	7.14 ± 0.36 c	19.05 ± 0.92 d
Abietinol	-1.88 ± 0.94 c	3.55 ± 0.63 f	21.10 ± 0.63 d
Prohydrojasmon	2.12 ± 0.85 b	4.34 ± 0.49 e	43.25 ± 0.50 c
Casbene	0.55 ± 0.49 c	1.42 ± 0.24 g	11.35 ± 0.60 e

The annotation of metabolites was based on comparison of accurate mass to the Scripps database (with 5 PPM of tolerance). Values are the means of three replicates ± SE; significance of difference was determined by one-way ANOVA followed by Tukey multiple comparisons test; the different letters within one column indicate statistically significant differences between the treatments (P < 0.05)

### Pot Experiment of Tobacco Black Shank Control

**Figure 7:**
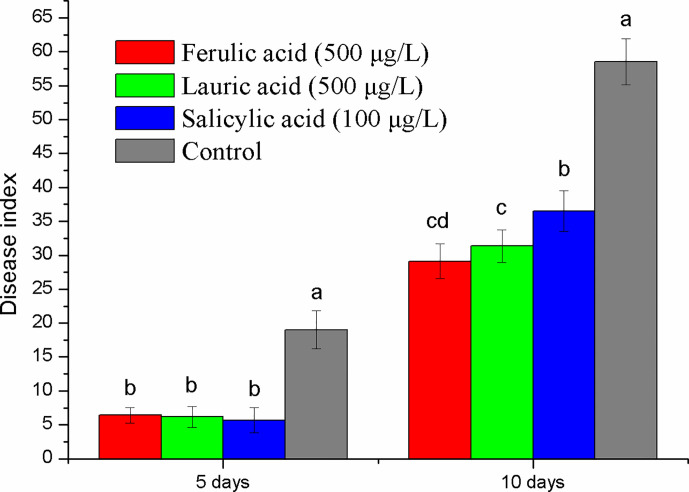
Disease control effect evaluation of the three compounds selected by pot experiment. Lower case letters indicate statistical differences (*P* < 0.05); the values are mean ± SE of three replicates for each treatment. Bars indicate SEs.

Ferulic acid and lauric acid were chosen for the disease control test because of their strong inhibitory activity. SA, the important disease resistance inducer, was also selected. The results of the pot experiment showed that all the compounds tested had a disease suppression effect (**Figure 7**). Compared with the control, the disease index of treatment with the three compounds was reduced by 63–70% and 27–58% at the fifth and tenth days after inoculation, respectively. The application of 500 µg ml^-1^ of ferulic acid and lauric acid showed better control efficacy than that of 100 µg ml^-1^ of SA.

## Discussion

### Effect of Root Exudates on *P. nicotianae*

Increasing evidence suggests that plant-pathogen interaction can be mediated by root exudates. Several studies have compared the allelopathic effects of root exudates on pathogens between disease-resistant and susceptible cultivars. Stevenson et al. (1995) reported that the root exudates of two wilt-resistant chickpea (*Cicer arietinum*) cultivars significantly inhibited the spore germination of *Fusarium oxysporum* f. sp. *ciceri* as well as the hyphal growth of the germinated spores, while another two susceptible cultivars showed no antifungal activity. Schalchli et al. (2012) found that there was a positive correlation between the root exudate activity on *Gaeumannomyces grainis* var. *tritici* and disease-resistance level of wheat (*Triticum aestivum*) varieties. Similar results have also been observed in other plant–pathogen interactions, including: eggplant (*Solanum melongena*)-*Verticillium dahliae* (Zhou et al., 2011), faba bean (*Vicia faba*)-*Fusarium oxysporum* f. sp. *fabae* (Dong et al., 2014), pepper (*Capsicum annuum*)-*Phytophthora capsici* (Wang et al., 2014), cotton (*Gossypium hirsutum*)-*F. oxysporum* f. sp. *vasinfactum* (Ren and Gai, 2016), and cotton-*V. dahliae* (Wu et al., 2007). In accordance with the previous studies, our results showed that root exudates of black shank-resistant tobacco cultivar Gexin 3 significantly inhibited the zoospore germination and mycelial growth of *P. nicotianae* compared to the control, whereas the root exudates of the susceptible cultivar Xiaohuangjin 1025 stimulated the colony growth but had no effect on spore germination.

However, there was no perfect correlation between root exudate activity and the overall level of disease resistance of crops, since the defense response after root infection is another important resistance mechanism. Bani et al. (2018) found that root exudates from three *Fusarium* wilt-resistant cultivars of pea (*Pisum sativum*) inhibited spore germination of *Fusarium oxysporum* f. sp. *pisi*, but the other seven resistant cultivars, as well as two susceptible cultivars, stimulated germination. However, there is no doubt that the inhibition effect of root exudates may limit the survival and infection of soil-borne pathogens in the rhizosphere. Therefore, root exudates constitute a pre-infection defense mechanism in many crops, and can be a potential indicator for predicting crop resistance to soil-borne diseases.

### Discriminant Compounds in Root Exudates

Organic acids were the most represented class of compounds in our findings. Several studies have reported that disease-resistant genotypes of crops are rich in phenolic acids (Sztejnberg et al., 1983; Bily et al., 2003; He, 2018). Jadhav et al. (2013) reported higher concentrations of ferulic acid in infected and non-infected resistant genotypes of castor (*Ricinus communis*), suggesting a critical role of phenols in castor disease resistance. Zhang et al. (2007) and Dong et al. (2014) found that the organic acid concentrations in pea root exudates showed a positive relation with disease resistance. In accordance with previous studies, higher concentrations of phenolic acids were also observed in root exudates of the resistant tobacco cultivar (R) than in those of the susceptible cultivar (S), suggesting important roles in disease resistance. SA is known as an important signal molecule for eliciting plant defenses, and especially systemic acquired resistance. Its up-regulation in root exudates of both Gexin 3 and Xiaohuangjin 1025 after *P. nicotianae* inoculation indicated that SA was involved in the plant defense response. Ferulic acid has also been reported to enhance plant disease resistance (Bily et al., 2003; He, 2018). Broad spectrum antifungal activity of organic acids (including ferulic acid and 6-hydroxyhexanoic acid) has been reported in previous studies (Ferrochio et al., 2013; Aranega-Bou et al., 2014; Ferruz et al., 2016). We also observed inhibitory effects of ferulic acid and tartaric acid against *P. nicotianae*. These results suggested that these organic acids in root exudates act not only to elicit defense responses in tobacco, but also directly inhibit *P. nicotianae* growth. Moreover, these functions may be more effective in the resistant cultivar Gexin 3 than in the susceptible cultivar Xiaohuangjin 1025.

There is increasing evidence that fatty acids are important in plant defense systems (Jiang et al., 2009; Kachroo and Kachroo, 2009). Linolenic and linoleic acid metabolites, such as oxylipins and jasmine acid, are known as signal molecules for systemic acquired resistance (SAR) (Blée, 2002). The unsaturated fatty acids, linolenic acid, linoleic acid, and oleic acid, have been reported to induce systemic resistance against *Phytophthora infestans* (Cohen et al., 1991). Increased levels of unsaturated fatty acids have been reported to enhance the resistance of tomato (*Lycopersicon esculentum*) against powdery mildew (*Erysiphe polygoni*) (Wang et al., 1998) and the resistance of eggplant against *V. dahliae* (Xing and Chin, 2000). In accordance with these observations, higher concentrations of linolenic acids in Gexin 3 root exudates indicated a possible correlation between linolenic acids and disease resistance. 6-hydroxyhexanoic acid is a derivative of hexanoic acid. This type of compound has been reported as a broad-spectrum inducer, acting mainly by enhancing the jasmonate signaling pathway (Vicedo et al., 2009; Llorens et al., 2013). 6-hydroxyhexanoic acid may play a similar role in Gexin 3, since higher content of 6-hydroxyhexanoic acid accumulated in root exudates of Gexin 3. Antifungal activity has also been reported for other fatty acids. For example, lauric acid exerts an inhibitory effect against *Aspergillus*, *Penicillium*, and *Fusarium* spp. (Altieri et al., 2009). The strong inhibitory activity of lauric acid was also observed in our study. These results indicated that fatty acids in root exudates may play a role in the plant defense response, not only by eliciting systemic resistance, but also by directly inhibiting the growth of pathogens.

Glycerol tripropanoate and isoamyl cinnamate were the main differential esters screened. Glycerol tripropanoate is mainly used in the manufacture of food, soaps, and candles, while the distribution and function of triglycerides in plants are not clear. Cinnamate and its derivative were ubiquitous in plant, but there are few studies of their roles in plant growth regulation (Shuab et al., 2016). In the current study, isoamyl cinnamate was extremely rich in root exudates of Xiaohuangjin 1025 compared to those of Gexin 3. Considering that it also showed no significant inhibitory (500 μg ml^-1^) or stimulatory effects on *P*. *nicotianae* (< 50 μg ml^-1^), isoamyl cinnamate may be involved in the susceptibility of Xiaohuangjin 1025. However, this proposition needs further experimental verification.

Alkaloids are a kind of secondary metabolites produced by plants and are believed to serve as defense compounds (Zhang et al., 2014). Most of the alkaloids annotated in the current study are reported to exist in tobacco and other plants (Saidkhodzhaeva and Bessonova, 1996; Xie and Kúc, 1997; Bessonova and Saidkhodzhaeva, 1998; Zhong et al., 2010), but were first found in tobacco root exudates. Rishitin is a terpenoid phytoalexin, and esculetin is a coumarin phytoalexin. The accumulation of these compounds is an important resistance mechanism against pathogen infection (Gutierrez et al., 1995; Komaraiah et al., 2003). In the current study, the accumulation of esculetin was enhanced by pathogen infection for both Gexin 3 and Xiaohuangjin 1025, while rishitin was only significantly increased in Xiaohuangjin 1025. The function of horhammericine, lycaconitine, olirotin, and nudicauline in plant has been little reported, and the accumulation of these compounds was not significantly affected (except for horhammericine in Gexin 3) by *P. nicotianae* infection. Overall, the higher base level of alkaloids in root exudates of Gexin 3 is not only genetically determined, but also closely related to disease resistance.

Plant endogenous hormones have significant effects on plant growth as well as disease resistance. Jasmonate is an important inducer of the plant defense system. As a derivative of jasmonate, prohydrojasmon has the same bioactivity (Koshiyama et al., 2006). Prohydrojasmon was detected in root exudates of Gexin 3, while it was minimal in root exudates of Xiaohuangjin 1025. These results indicated that the jasmonate signal pathway was efficient in Gexin 3, but may be blocked in Xiaohuangjin 1025.

### Possible Disease Resistance Signal Pathway Associated With Root Exudates

**Figure 8:**
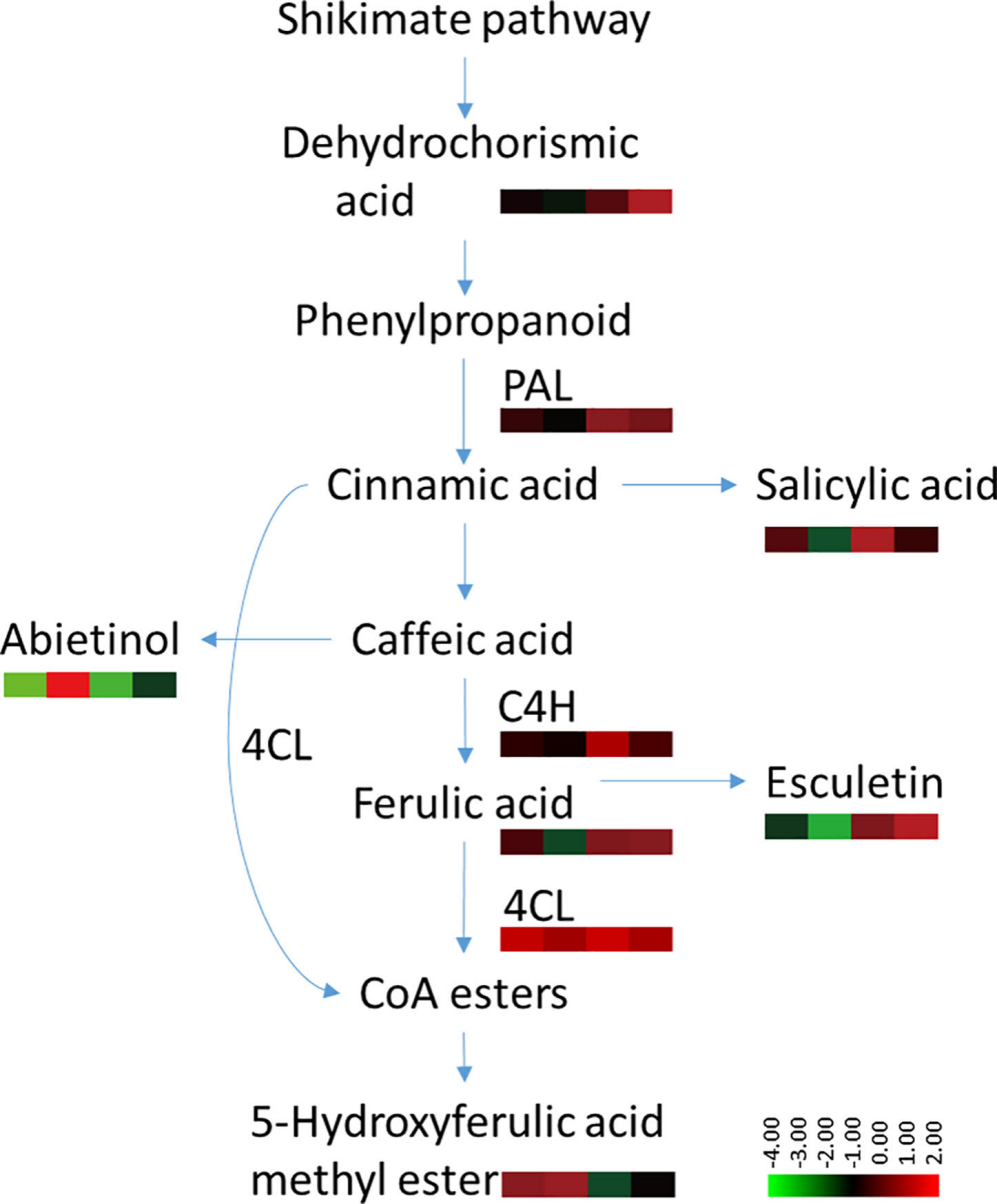
Variable content and enzyme activities associated with the phenylpropanoid metabolism pathway in tobacco*-P. nicotianae* interaction. The annotation of metabolites was based on comparison of accurate mass to the Scripps database (with 5 PPM of tolerance). The color scale represents the relative compound abundance and enzyme activities; columns represent the four treatments from left to right: non-inoculated Gexin 3 (R), non-inoculated Xiaohuangjin 1025 (S), inoculated Gexin 3 (Ri), and inoculated Xiaohuangjin 1025 (Si). PAL, phenylalanine ammonia-lyase; C4H, cinnamate-4-hydroxylase; 4CL, 4-coumarate-CoA ligase; CoA, coenzyme A. The enzyme activity data were obtained after 3 days of pathogen inoculation.

The phenylpropanoid metabolism pathway plays an important role in plant disease resistance and defense responses (Hammerschmidt, 1999), and the main defensive compounds, such as phenolics, phytoalexin, lignin, and flavonoids, need to be synthesized through this pathway (Jadhav et al., 2013). Pathogen infection usually activates phenylpropane metabolism in plants to enhance host resistance (Gong et al., 1995; Slatnar et al., 2010). There is considerable evidence to indicate that the phenylpropanoid metabolic activity of resistant varieties is significantly higher than that of susceptible varieties (Long et al., 2004; Wang et al., 2009; Bao et al., 2015). Several discriminant compounds are associated with the phenylpropanoid metabolism pathway, including SA, ferulic acid, esculetin and methyl 5-hydroxyferulate. The higher expression of these phenylpropanoid compounds in root exudates of both infected and uninfected plants indicated a higher basal and induced activity of phenylpropanoid metabolism pathway in the resistant cultivar Gexin 3 compared to the susceptible cultivar Xiaohuangjin 1025 (**Figure 8**). These suggestions were partially confirmed by key enzyme activity assays associated with the phenylpropanoid metabolism pathway. PAL is the key and rate limiting enzyme in phenylpropanoid metabolism, which is usually used to evaluate the resistance of plants (Stadnik and Buchenauer, 2000). C4H is associated with the synthesis of coumaric acid, the precursor for caffeic and ferulic acid synthesis, which can directly inhibit the growth of pathogens. 4CL catalyzes the formation of phenols, flavonoids, lignin, and other metabolites, which are believed to be important defense substances in plants. The higher activities of PAL, C4H, and 4CL in Gexin 3 may improve the synthesis and accumulation of inhibitory substances such as ferulic acid, which were in line with the root exudate profiling results. In accordance with previous studies (Gong et al., 1995; Slatnar et al., 2010), the higher activities of PAL, C4H, and 4CL were also observed in the roots of Gexin 3. These results indicated the higher phenylpropanoid metabolic activity in Gexin 3 may be associated with the secretion of antifungal substances to the rhizosphere.

### Inhibitory Activity of the Discriminant Compounds on *P. nicotianae* In Vitro and In Vivo

Some discriminant compounds in tobacco root exudates showed a strong inhibitory effect against *P. nicotianae* mycelial growth, which supports the suggestion that root exudates can mediate plant disease resistance by direct inhibition of pathogens. Among them, tartaric acid and ferulic acid showed strong inhibition effect on *P. nicotianae* growth as well as disease suppression. The disease control effect has been reported in previous studies including tobacco black shank (Zhang and Wang, 2006; Fu et al., 2013; Feng et al., 2018). He (2018) reported that ferulic acid can induce resistance to gray mold (*Botrytis cinerea*) in apples. Our results also showed that SA significantly suppressed tobacco black shank by induced resistance, since it showed no inhibitory effect on *P. nicotianae* at test concentrations. Our previous study has proved that hydrojasmon has certain disease prevention effects on tobacco black shank (Feng et al., 2018). The above evidence indicated root exudates can be a source of disease resistant substances, and tartaric acid, ferulic acid, lauric acid, SA, and hydrojasmon have potential for use in tobacco black shank control.

## Conclusions

The current study revealed differing metabolic patterns and functions between root exudates of a disease-resistant tobacco cultivar and a susceptible tobacco cultivar. Root exudates not only provide a pre-infection prevention strategy for tobacco by exuding antimicrobial substances to directly inhibit *P. nicotianae* growth, but also increase tobacco disease resistance by eliciting plant defense responses. Compared to the susceptible cultivar Xiaohuangjin 1025, the resistant cultivar Gexin 3 has higher richness of defensive compounds in root exudates. Our results provide useful insights into possible disease resistance mechanisms of root exudates, and attempt a beneficial utilization of these secondary metabolites of plants.

It should be noted that some inferences in this study need further experimental verification (i.e., the signal pathway related to defense responses). In addition, root exudates can also affect the microbial community in the rhizosphere, which is worthy of further study.

## Data Availability Statement

The original contributions presented in the study are included in the article/**Supplementary Material**; further inquiries can be directed to the corresponding authors.

## Author Contributions

CZ and FW contributed to the conception and design of the study. CZ wrote the first draft of the manuscript. CF and JW performed the investigation. YZ performed the statistical analysis.

## Conflict of Interest

The authors declare that the research was conducted in the absence of any commercial or financial relationships that could be construed as a potential conflict of interest.
