# Supplementary material for: Root Exudates Metabolic Profiling Suggests Distinct Defense Mechanisms Between Resistant and Susceptible Tobacco Cultivars Against Black Shank Disease
Source: Front Plant Sci. 2020 Sep 10;11:559775. doi: 10.3389/fpls.2020.559775 (PMC7511587; doi:10.3389/fpls.2020.559775)

Supplementary for

Root Exudates Metabolic Profiling Suggests Distinct Defense Mechanisms Between Resistant and Susceptible Tobacco Cultivars Against Black Shank Disease

Chengsheng Zhang^1, 2, 3#*^, Chao Feng ^1,3#^, Yanfen Zheng^1^, Jing Wang^1, 2^, Fenglong Wang^1,3*^

^1^Marine Agriculture Research Center, Tobacco Research Institute of Chinese Academy of Agricultural Sciences, Qingdao, China

^2^Qingdao Special Crops Research Center of Chinese Academy of Agricultural Sciences, Qingdao, China

^3^Pest Integrated Management Key Laboratory of China Tobacco, Qingdao, China

Running title: Metabolic profiling of tobacco root exudates

# Authors contributed equally to the article.

***Correspondence:**
Fenglong Wang

[wangfenglong@caas.cn](mailto:wangfenglong@caas.cn)

Chengsheng Zhang

[zhangchengsheng@caas.cn](mailto:zhangchengsheng@caas.cn)

^#^These authors contributed equally to this work.

Keywords: *Phytophthora nicotianae,* metabolic profiling; tobacco; *Nicotiana*; disease resistance; defense mechanisms

Figure S1 The disease incidence of different varieties after 7 days of *Photophthora nicotianae* inoculation. a, inoculated Xiaohuangjin 1025; b, inoculated Gexin 3; c, non-inoculated Xiaohuangjin 1025; d, non-inoculated Gexin 3.

**
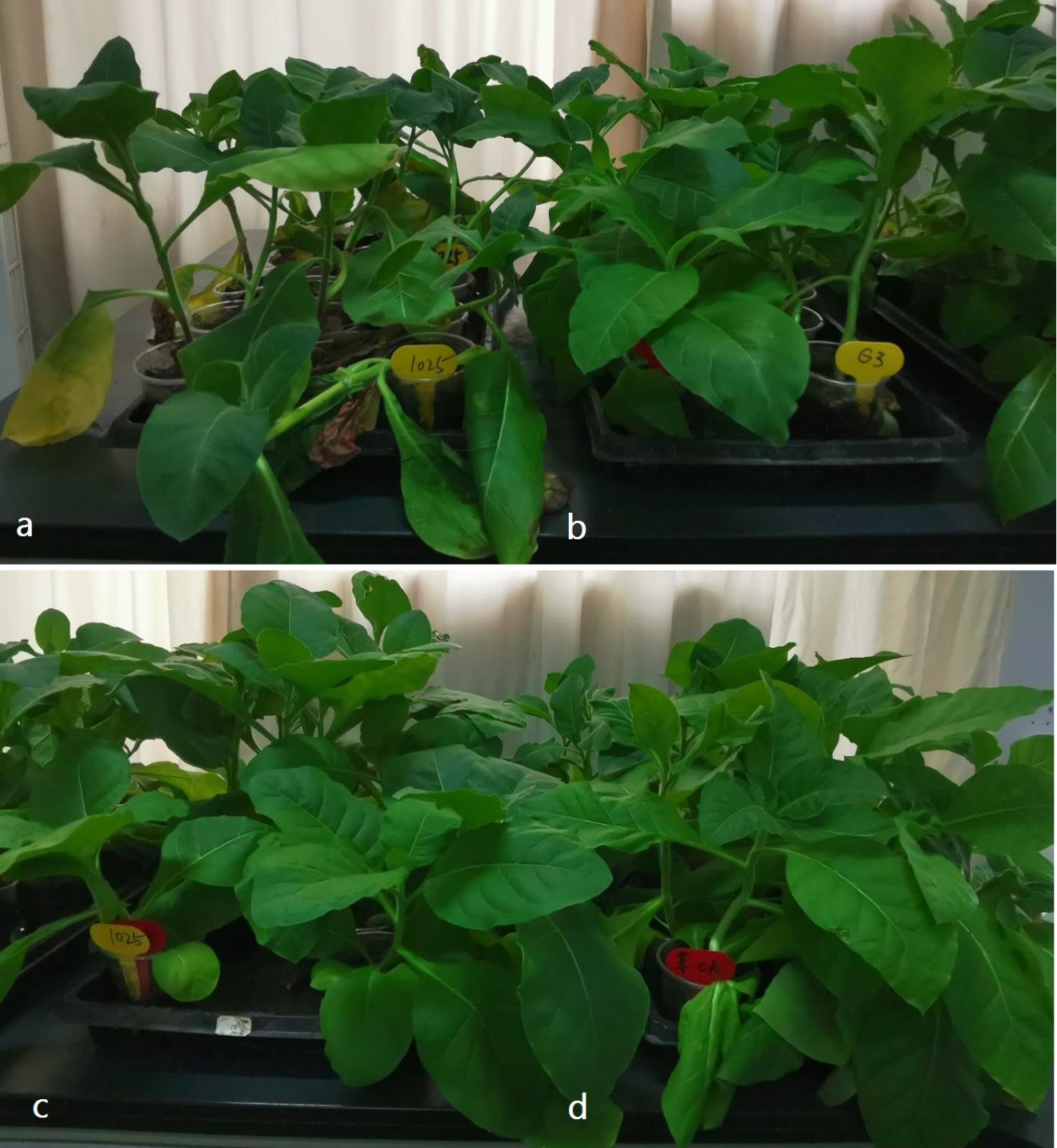
**

**Figure S2** Total ions current overlaps of the quality control (QC) samplesby mass spectrometry detection. a, six samples at the beginning, one QC sample after the first six samples, and one QC sample after the last six samples under ESI positive mode; b, the first and last QC samples under ESI negative mode.


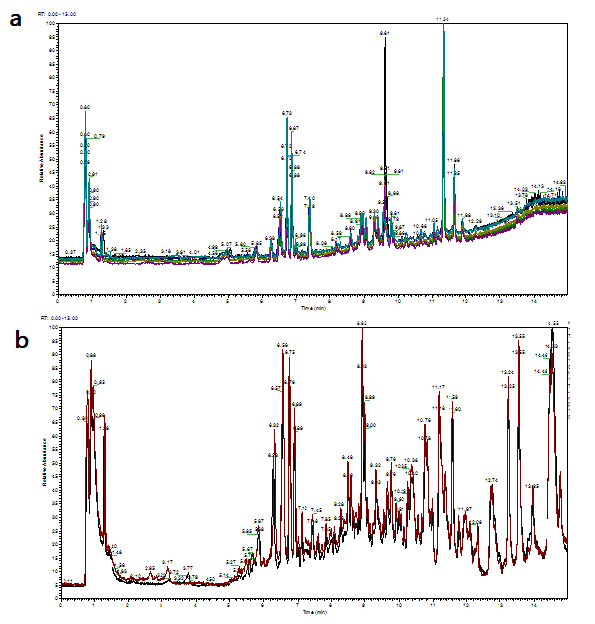


**Table S2.** Gibbs free energies*^a^* and equilibrium populations*^b^* of low-energy conformers of **3**.

| Conformers | In MeOH | |
| --- | --- | --- |
|  | *G* | *P* (%) |
| **3-1** | -1181751.9763257 | 18.35 |
| **3-2** | -1181751.88784679 | 15.80 |
| **3-3** | -1181752.12504557 | 23.59 |
| **3-4** | -1181752.13634075 | 24.04 |
| **3-5** | -1181751.97193313 | 18.21 |
| *^a^*B3LYP/6-31+G(d,p), in kcal/mol. *^b^*From *G* values at 298.15K. | | |

**Figure S3 Heatmap of the Top 22 discriminant metabolites in tobacco root exudates. The data were normalized with values of Log (peak area of each compound /average peak area of each compound within four groups). R, non-inoculated Gexin 3; S, non-inoculated Xiaohuangjin 1025; Ri, inoculated Gexin 3; Si, inoculated Xiaohuangjin 1025.**


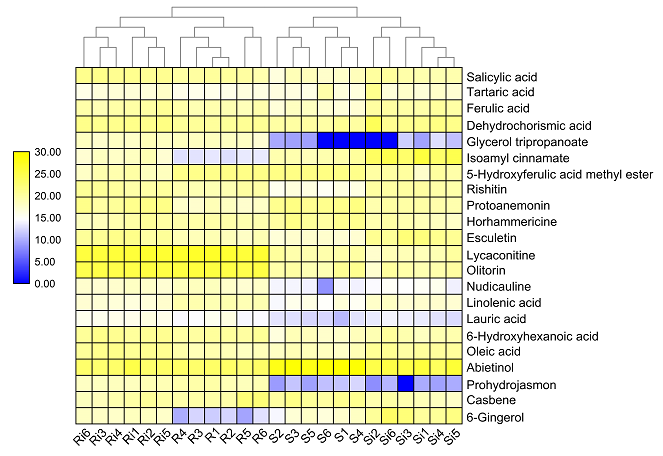

Supplement: Supplementary file 1 [file Table_1.docx]
